# Effectiveness of skin-to-skin contact versus care-as-usual in mothers and their full-term infants: study protocol for a parallel-group randomized controlled trial

**DOI:** 10.1186/s12887-017-0906-9

**Published:** 2017-07-06

**Authors:** Kelly H. M. Cooijmans, Roseriet Beijers, Anne C. Rovers, Carolina de Weerth

**Affiliations:** Department of Developmental Psychology, Behavioural Science Institute, Radboud University, P.O. Box 9140, 6500 HE Nijmegen, The Netherlands

**Keywords:** Skin-to-skin contact, Full-term infants, Postpartum depressive symptoms, Mental health, Physical health, Mother-infant relationship, Infant behavior, Randomized controlled trial

## Abstract

**Background:**

Twenty-to-forty percent of women experience postpartum depressive symptoms, which can affect both the mother and infant. In preterm infants, daily skin-to-skin contact (SSC) between the mother and her infant has been shown to decrease maternal postpartum depressive symptoms. In full-term infants, only two studies investigated SSC effects on maternal depressive symptoms and found similar results. Research in preterm infants also showed that SSC improves other mental and physical health outcomes of the mother and the infant, and improves the quality of mother-infant relationship. This randomized controlled trial will investigate the effects of a SSC intervention on maternal postpartum depressive symptoms and additional outcomes in mothers and their full-term infants. Moreover, two potential underlying mechanisms for the relation between SSC and the maternal and infant outcomes will be examined, namely maternal oxytocin concentrations and infant intestinal microbiota.

**Methods/design:**

*Design:* A parallel-group randomized controlled trial. *Participants*: 116 mothers and their full-term infants. *Intervention:* Mothers in the SSC condition will be requested to provide daily at least one continuous hour of SSC to their infant. The intervention starts immediately after birth and lasts for 5 weeks. Mothers in the control condition will not be requested to provide SSC. Maternal and infant outcomes will be measured at 2 weeks, 5 weeks, 12 weeks and 1 year after birth. *Primary outcome:* maternal postpartum depressive symptoms. *Secondary maternal outcomes:* mental health (anxiety, stress, traumatic stress following child birth, sleep quality), physical health (physical recovery from the delivery, health, breastfeeding, physiological stress), mother-infant relationship (mother-infant bond, quality of maternal caregiving behavior). *Secondary infant outcomes*: behavior (fussing and crying, sleep quality), physical health (growth and health, physiological stress), general development (regulation capacities, social-emotional capacities, language, cognitive and motor capacities). *Secondary underlying mechanisms*: maternal oxytocin concentrations, infant intestinal microbiota.

**Discussion:**

As a simple and cost-effective intervention, SSC may benefit both the mother and her full-term infant in the short-and long-term. Additionally, if SSC is shown to be effective in low-risk mother-infant dyads, then thought could be given to developing programs in high-risk samples and using SSC in a preventive manner.

**Trial registration:**

NTR5697; Registered on March 13, 2016.

**Electronic supplementary material:**

The online version of this article (doi:10.1186/s12887-017-0906-9) contains supplementary material, which is available to authorized users.

## Background

Having a new baby is an extraordinary experience that most often brings much joy and happiness. At the same time, the first months after birth can also be challenging for parents. A large number of mothers experience postpartum depressive symptoms, which can affect both the mother and the infant. In preterm infants, daily skin-to-skin contact (SSC) between the mother and her infant has been shown to decrease maternal postpartum depressive symptoms. In full-term infants, only two studies investigated the effectiveness of SSC on maternal postpartum depressive symptoms and found similar results. Studies in preterm infants showed that SSC improves other mental and physical health outcomes in mothers as well as the infants, and promotes the quality of mother-infant relationship. Based on these previous findings, it can be hypothesized that SSC may have additional positive effects in mothers and their full-term infants as well. This randomized controlled trial (RCT) will be the first to investigate the effectiveness of a SSC intervention in decreasing maternal postpartum depressive symptoms and improving additional outcomes in mothers and their full-term infants, including maternal mental health, maternal and infant physical health, infant crying and sleeping behavior, the quality of the mother-infant relationship, and the long-term infant general development even up to 1 year of age. Additionally, two potential underlying mechanisms for the effectiveness of SSC on maternal and infant outcomes will be examined, namely maternal oxytocin concentrations and infant intestinal microbiota.

### Maternal postpartum depressive symptoms

Between 20 to 40% of mothers show subclinical levels of depressive symptoms within the postpartum period [1–3]. Compared to mothers without depressive symptoms, these mothers tend to be more irritable and show less maternal warmth and sensitivity to their newborn [4]. In turn, this can disturb the mother-child interaction, which in the long-term has been related to multiple negative child outcomes such as poorer emotional and cognitive development [4–6]. Moreover, mothers who have experienced postpartum depressive symptoms in the past, are significantly more likely to experience recurrent depressive symptoms even up to 4 years after birth [7]. Especially, these recurrent depressive symptoms seem to be related to negative child outcomes [6].

A wide range of prevention and intervention programs are available for mothers who experience postpartum depression, including pharmaceuticals and psychotherapy. The results with regard to the effectiveness are mixed [4, 8]. In addition, postpartum depression often remains undetected or remains untreated for a long period of time [9, 10]. Most mothers with subclinical levels of depressive symptoms will often not receive any treatment at all. Ideally, it would be best to prevent the mother from having depression and depressive symptoms. Hence, there is a great need for low-cost and easy-to-implement prevention or (complementary) intervention programs that are accessible for every mother, and that can be commenced immediately after birth.

### Skin-to-skin contact

Daily SSC between the mother and the infant is a promising, simple, and natural intervention that may be effective in decreasing postpartum depressive symptoms in mothers. During SSC, the naked infant, wearing only a diaper, is placed onto the bare chest of the mother [11]. The SSC method was designed as an alternative treatment to incubators for low birth weight and preterm infants.

In preterm infants, multiple studies showed that daily SSC results in lower levels of maternal depressive symptoms [12, 13]. To date, it is mainly unknown whether this positive effect extends to mothers of full-term infants. In a case study, a mother with numerous indicators of postpartum depressive symptoms was encouraged to provide SSC to her full-term infant. Daily SSC in the first days after birth reduced the levels of reported depressive symptoms of this mother and the symptoms remained absent even up to 7 months after birth [14]. Another study in full-term infants conducted a one-month SSC intervention trial starting immediately after birth [15]. Mothers were encouraged to provide 6 h of SSC with their infant on a daily basis during the first week after birth, and then 2 h per day until the infant was 1 month of age. This study showed that mothers in the intervention group (*n* = 30), compared to mothers in the control group (*n* = 60), showed statistically significant lower levels of postpartum depressive symptoms at 1 week and at 1 month after birth. These results are promising, however, the studies included relatively small sample sizes and did not perform a RCT. In the current RCT, as a primary outcome, we will investigate whether a 5-week SSC intervention period starting at birth reduces depressive symptoms up to 1 year postpartum in mothers of full-term infants.

### Additional effects of SSC in mothers and their full-term infants

In addition to the possible effectiveness of SSC on maternal postpartum depressive symptoms, SSC may also be related to other positive effects for the mother. In studies with preterm infants, research has shown that daily SSC is associated with lower levels of maternal reported anxiety and stress [16]. Also, SSC is reported to facilitate positive feelings in the mother and enhance her bonding to the infant, which in turn improves the quality of maternal caregiving behavior (e.g. more sensitive and less intrusive caregiving behavior) [16, 17]. Lastly, SSC in preterm infants improved the duration of breastfeeding [18].

In mothers of full-term infants, SSC during the first hours after birth decreases maternal anxiety, improves maternal caregiving behavior and is associated with longer breastfeeding duration [19, 20]. Only one study studied the effects of SSC on maternal outcomes beyond the first postnatal hours. Next to maternal postpartum depressive symptoms, this study found that a one-month intervention period of SSC was associated with a quicker maternal postpartum decrease in cortisol concentrations [15]. Cortisol can be described as a measure of physiological stress, since cortisol is the end product of the hypothalamic-pituitary-adrenal axis that is activated in response to stressful events [21].

Previous research in adults has shown that relaxation interventions can improve sleep quality, physical health, and recovery after surgery [22–24]. Moreover, multiple reviews show that touch, including physical intimacy and massage therapy, decreases physiological stress and pain and improves sleep and health [25, 26]. As SSC may constitute a daily hour of maternal relaxation that also includes touch, SSC may also improve maternal sleep quality, physical health, and physical recovery from the delivery.

The potential positive effects of SSC are not restricted to the mothers, as SSC may also be related to various beneficial outcomes in the full-term infant. In preterm infants, daily SSC has been related to better infant health and reduced stress, including increased weight gain, fewer infections, less crying, better sleep and attenuated cortisol stress responses [16, 27, 28]. Long-term effects of SSC are also seen, as SSC in preterm infants is related to better cognitive and motor development at 6 months of age and even to better cognitive control at age ten [13, 16].

In full-term infants, a systematic review showed that SSC immediately after birth for the first postnatal hours was related to less crying and fussing [19]. SSC also seems to have a protective effect during medical procedures. Full-term infants who were in SSC during a heel lance procedure cried less [29]. Also, 5-to-6 week old full-term infants’ cortisol concentrations decreased when resting in SSC, when compared to resting alone in a crib or playpen [30]. Another study compared sleep quality in 2-day old infants sleeping alone in a crib next to the mother or sleeping in SSC with their mother. Infants in SSC slept for a longer period of time, spent most of the time in quiet sleep and needed a shorter period to enter quiet sleep than when they were sleeping in a crib next to the mother [31]. In the current study, as secondary outcomes, we will investigate whether a 5-week daily SSC intervention period starting at birth improves maternal mental health, maternal and infant physical health, infant crying and sleeping behavior, the quality of the mother-infant relationship, and the long-term infant general development up to 1 year of age.

### Potential underlying mechanisms

The underlying working mechanisms that relate SSC early in life to improved maternal and infant outcomes are mainly unknown. One possible underlying mechanism is maternal oxytocin concentrations. The hormone oxytocin is released after the activation of multiple sensory nerves and in response to low intensity stimulation of the skin, including touch, as well as to suckling of the nipple [32, 33]. Oxytocin concentrations increase during social interaction, including parent-infant interactions [34]. The elevated concentrations of oxytocin are related to improved maternal mental health, by means of lower levels of maternal anxiety and stress, and improved bonding between mother and child [35]. To conclude, SSC may increase the maternal concentrations of oxytocin through the activation of multiple sensory nerves. In turn, these increased concentrations of oxytocin may explain the improved maternal mental health, the better quality of maternal caregiving behavior, and the longer duration and higher frequency of breastfeeding.

Another possible underlying mechanism of SSC is the infant intestinal microbiota. Infants are born with virtually sterile intestines. In vaginal deliveries, colonization of bacteria in the intestines starts during birth with bacteria originating mainly from the mother [36]. The intestinal ecosystem or microbiota consists mainly of bacteria and rapidly matures in the first months of life [37]. This early microbial colonization is important for the development of the immune system and the gastrointestinal tract [37, 38]. Less stable or diverse intestinal microbiota may delay the development of the immune system and is associated with diseases and excessive crying in the infant [39, 40].

Potentially, SSC facilitates the development of the infant intestinal microbiota, subsequently influencing infant outcomes, through direct and indirect pathways. First, daily periods of SSC could provide extra opportunities to exchange bacteria from the mother’s skin to the infant, hence enhancing microbiota development. Research has shown that infants born with a C-section receive fewer of their mother’s bacteria, but bacteria can still be passed on from the skin of the mother to the infant although in a lower frequency [41]. Second, as already mentioned above, SSC potentially reduces maternal depressive symptoms, anxiety, and reported and physiological stress levels in the mother. The improved maternal mental health may positively affect her quality of breast milk, which in turn is important for the healthy maturation of the infant intestinal microbiota during the first year of life [42]. Third, research has shown that there is a bidirectional communication between the gut and the brain, in which the stress hormone cortisol plays an important role [43]. In animal models, stress experienced by an organism can modify the balance of the gut microbiota, making the path clear for colonization by pathogenic bacteria, which in turn may affect immune and brain functioning [44]. SSC may be related to less stress in the infant, which in turn may facilitate the optimal colonization of the intestines of the infant. In sum, there are multiple ways through which an improved infant intestinal microbiota may mediate the relation between SSC and positive infant outcomes.

### Objectives

The primary goal of this RCT, with two parallel groups (intervention versus passive control group), is to examine if daily mother-infant SSC is effective in decreasing (sub)clinical levels of maternal depressive symptoms in a non-clinical sample with full-term infants in comparison to mothers who care for their infant as usual. The intervention starts immediately after delivery and has a duration of 5 weeks. The secondary aim is to investigate additional maternal and full-term infant outcomes, and two possible underlying mechanisms: maternal oxytocin concentrations and infant intestinal microbiota. We hypothesize that, compared to the control (care-as-usual) group, the SSC group will show:

*Improved maternal outcomes:*

1. Mental health:

- lower levels of depressive symptoms (primary outcome);
- lower levels of anxiety;
- lower levels of stress;
- lower levels of traumatic stress following child birth;
- better sleep quality.

2. Physical health:

- better physical recovery from the delivery;
- better health (fewer illnesses and health problems);
- more frequent and a longer duration of breastfeeding;
- lower levels of physiological stress.

3. Mother-infant relationship:

- better bonding to the infant;
- better quality of maternal caregiving behavior.

*Improved infant outcomes:*

1. Behavior:

- lower amounts of daily fussing and crying;
- better sleep quality.

2. Physical health:

- better growth and health (fewer illnesses and health problems);
- lower levels of physiological stress.

3. General development:

- better regulation capacities;
- better social-emotional capacities;
- better language, cognitive, and motor capacities.

*Potential underlying mechanisms:*

1. higher levels of maternal oxytocin concentrations. Maternal oxytocin concentrations will mediate the relationship between SSC and maternal outcomes.
2. more optimal levels of intestinal microbiota (i.e. faster developing microbiota, more diverse and more stable microbiota, and fewer potentially pathogenic bacteria). Infant intestinal microbiota will mediate the relationship between SSC and infant outcomes.

## Methods

### Study design

This SSC intervention project in mothers and their full-term infants is a RCT with two parallel groups (intervention versus passive control group). The study has been designed in accordance with the SPIRIT 2013 statement (see Additional file 1 for complete checklist) [45]. Administrative information related to the trial can be obtained in Additional file 2. The project will start in April 2016. The expected total duration of the study from the start of recruitment to the last participant finishing the 1-year assessment is 20 months. The study will start with baseline measures in the last trimester of pregnancy. The intervention period will start immediately after birth and will have a duration of 5 weeks. Maternal and infant outcomes will be measured at 2 weeks, 5 weeks, 12 weeks and 1 year after birth.

### Ethics

The trial has been approved by the ethics committee of the Social Science faculty of the Radboud University in Nijmegen, The Netherlands (ECSW2015–2311-358). Modifications to the protocol, including changes of study objectives, study design, population, sample sizes, study procedures, or significant administrative aspects will require a formal amendment to the protocol. The potential amendment will be examined and approved by the ethics committee of the Social Science faculty of the Radboud University prior to implementation. Modifications to the protocol will also be included in the formal trial registration at the Dutch Trial Registration (NTR). This study does not involve any harmful procedures or adverse events. Since trial-related harm is negligible no provisions will be included, the study will not be audited, and no data-monitoring committee will be needed. Although we do not expect that the participants will experience trial-related harms, mothers will be asked weekly, for the first 5 weeks after birth, about any problems and mothers will be encouraged to report harms or adverse events spontaneously. Also, during the home visit in week 5 we will ask mothers to report any previously unreported harmful or adverse events that occurred during the study trial. Answers will be assessed by the investigators and will be reported in study reports. In addition, all participants that are enrolled in the study will be covered by the indemnity for negligent harm insurance (standard arrangements of the Behavioural Science Institute, Radboud University, Nijmegen, The Netherlands).

### Sample size calculation

The sample size was calculated with a power analysis, using the G Power 3 online tool [46]. The effect size of f = .24 for maternal depressive symptoms was based on the study of Bigelow and colleagues and was calculated by using the mean levels and the pooled standard deviation of maternal depressive symptoms for the SSC and the control group [15]. The power analysis indicated that a total group size of 84 mother-infant dyads was required to maintain a power of 80% to detect a minimum effect size of f = .24 for maternal depressive symptoms with a significance level of 0.05. Based on a prior longitudinal study in a similar population [47], we assume that 14% of the mothers will sign up to the study but will never participate. Based on the same longitudinal study [47], we calculated that the drop-out rate will be 4% and the loss in follow up rate will be 9%. Additionally, we estimate a non-compliance rate of 10%. In total, we need to include 31 additional pregnant women in order to retain power. To maintain equal groups we will recruit 116 participants (58 mothers and their infants in each arm).

### Participants

**Fig. 1:**
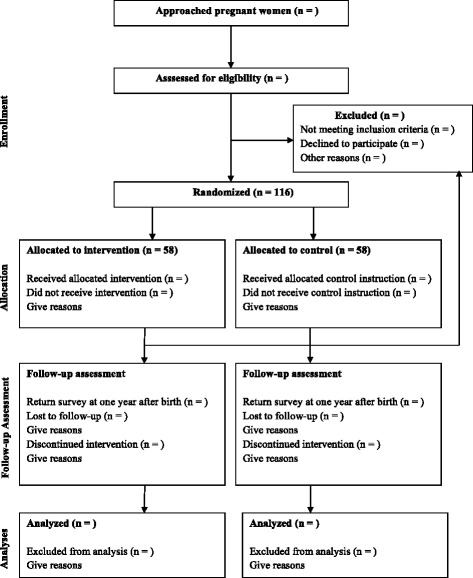
Participant flow

A total sample of 116 healthy expectant mothers will be recruited during pregnancy in the region of the Radboud University, The Netherlands. In this region, 17.800 children are born every year [48]. Pregnant women have to meet the following eligibility criteria: age ≥ 18, singleton pregnancy, no drug use during pregnancy, no severe physical or mental health problems, and sufficient understanding of the Dutch language. Also, exclusive participation in this intervention trial is required. The infants have to be born at ≥37 weeks of pregnancy and have to meet the following eligibility criteria: no congenital anomalies, birth weight ≥ 2500 g, and 5-min Apgar score ≥ 7. The participant flow is displayed in Fig. 1.

### Outcomes

Tables 1 and 2 show the maternal and infant primary and secondary outcomes at the different time points. Table 3 shows the additional information, such as the demographics and confounders that will be collected in the current study.

**Table 1:** Maternal outcome measures

Construct	Timing (W = week/Y = Year)				Measure	Additional information
	W2	W5	W12	Y1		
Primary outcome						
1. Mental health						
1.1 Depressive symptoms	x	x	x	x	Edinburgh Postnatal Depression Scale (EPDS)	10-item validated questionnaire to screen for depressive symptoms in a post-partum setting [53].
Secondary outcomes						
1. Mental health						
1.2 Anxiety	x	x	x	x	State-Trait Anxiety Inventory (STAI)	The STAI is a 40-item validated questionnaire to screen for anxiety [54].
1.3 Stress	x	x	x	x	Alledaagse Problemen Lijst (APL)	The APL is a 49-item validated questionnaire to assess the occurrence and intensity of daily hassles [55].
1.4 Traumatic Stress following child birth	x	x	x	x	Traumatic Event Scale-B (TES-B)	The TES-B is a 35-item validated questionnaire to examine traumatic stress after giving birth to a child [56].
1.5 Sleep quality	x	x	x	x	Adjusted version of the Karolinska Sleep Diary (KSD) & Pittsburgh Sleep Quality Index (PSQI)	Every morning, for three consecutive days, the mother will fill in the KSD starting at week two, five and twelve [57]. In addition, questions related to daytime sleep and reasons for wakening will be included. At twelve weeks and one year, the mother will fill in de PSQI [58]. The PSQI is a 19-item validated questionnaire to examine sleep quality and disturbances in reference to their sleep during the past month.
2. Physical health						
2.1 Physical recovery related to the delivery	x	x	x	x	Maternity carer logbook & Multidimensional Fatigue Inventory (MFI) & Bodily Pain subscale of the Short-Form Health Survey (SF-36)	The maternal physical recovery logbook of the maternity carer from the first week after birth will be copied at the second home visit. The MFI is a 20-item validated questionnaire to examine fatigue [59]. The two-item subscale of the SF-36 will be used to examine bodily pain [60].
2.2 Health		x	x	x	Health interview & questionnaire	A maternal health interview, based on the infant health interview [61], will be performed during the second home visit. During the follow-up assessment the mother will fill out health questionnaires, based on the infant health interview [61].
2.3 Breastfeeding	x(w1–12)			x	Weekly logbook	The mother will fill out a weekly logbook including the estimated times of breastfeeding, expressed breastmilk feeding, and formula feeding to examine breastmilk initiation, exclusivity, frequency and duration. At one year of age, mothers will note the number of months the infant was breastfed and she will note the current feeding status.
2.4 Physiological stress		x			Hair cortisol	Hair analyses will be used to show the activity of the HPA axis over longer periods of time. A strand of hair will be cut from the scalp.
3. Mother-infant relationship						
3.1 Mother-infant bond	x	x	x	x	Maternal Postnatal Attachment Scale (MPAS)	The MPAS is a 19-item validated questionnaire to assess current maternal attitudes, thoughts, and feelings towards the child [62].
3.2 Quality of maternal caregiving behavior (sensitivity)		x			Ainsworth Sensitivity Scales	An infant bathing session during the home visit on week five will be videotaped. After, reliable observers, blinded for group allocation, will observe maternal sensitivity [63].
3.3 Quality of maternal caregiving behavior (synchrony)		x			Saliva cortisol	Synchrony will be measured with saliva samples of both the mother and infant before and after the bathing session (undressing, bathing and dressing). Samples will be collected right before undressing (baseline, T1), and 25 (stress, T2) and 40 (recovery, T3) minutes after the infant is taken out of bath [64]. Saliva will be collected in tubes for the mother and with eye sponges for the child [65]. Cortisol synchrony consists of the within mother-infant dyad cortisol associations (i.e. mother cortisol predicting infant cortisol, infant cortisol predicting mother cortisol). Cortisol synchrony will be examined for every saliva sample (pre-stress, peak-stress, recovery), for total stress cortisol concentrations (Area Under the Curve (AUC)), and cortisol recovery (recovery – pre-stress).
Secondary outcomes: Underlying mechanism						
Oxytocin concentrations		x			Saliva oxytocin	Maternal oxytocin concentrations during the bathing session will be measured in saliva at the same time points as cortisol. Saliva will be collected in tubes.

**Table 2:** Infant outcome measures

Construct	Timing (W = week/Y = Year)				Measure	Additional information
	W2	W5	W12	Y1		
Secondary outcomes						
1. Behavior						
1.1 Crying and fussing	x	x	x		72-h study diary	Crying and fussing will be collected with a simply designed 72-h logbook that mothers fill in with lines [66].
1.2 Sleep quality	x	x	x	x	72-h study diary & Adjusted version Brief Infant Sleep Questionnaire (BISQ)	Mother will note the amount of sleep time in the crying and fussing diary at week two, week five, and week twelve [66]. Additionally, the mothers will complete the infant sleep-screening tool in reference to their child’s sleep during the past week at week two, week five, week twelve and year one [67].
2. Physical health						
2.1 Growth & health		x	x	x	Well-baby clinic logbook & Health interview and questionnaires	Growth and weight information will be copied from the well-baby clinic logbook at week 12 and year one. In addition, a health interview will be performed during the second home visit [61]. Also, the mother will fill in health questionnaires, based on the infant health interview, at week 12 and at 1 year of age [61].
2.2 Physiological stress		x			Saliva cortisol	Infant cortisol reactivity and recovery will be measured with the saliva samples of the infant during the bathing session [64].
3. General development						
3.1 Regulation capacities			x	x	Infant Behaviour Questionnaire Revised (IBQ) – Short form	The IBQ is a 91-item questionnaire to assess infant regulation capacities with the Orienting/Regulation subscale [68].
3.2 Language, cognitive and motor capacities				x	Ages and Stages Questionnaire- Third edition (ASQ-3)	The ASQ-3 is a 30-item validated questionnaire to assess communication, gross motor, fine motor, and adaptive problem solving skills [69]. The Personal-Social subscale of the ASQ will be used as a measure for social-emotional capacities (see 3.3).
3.3 Social-Emotional capacities				x	Brief Infant Toddler Social Emotional Assessment (BITSEA)	The BITSEA is a 42-item validated questionnaire to examine social and emotional behavior such as externalizing and internalizing behavior [70]. The Personal-Social subscale of the ASQ will be used (see 3.2) [69].
Secondary outcomes-Underlying mechanism						
Intestinal microbiota	x	x		x	Stool samples	Stool will be collected from the diaper by the parents. Stool will be collected in tubes.

**Table 3:** Additional information

Measure	Timing (P = late pregnancy/W = week/Y = Year)					Details
	P	W2	W5	W12	Y1	
Eligibility criteria						
Medical checklist	x					During the telephone call in pregnancy the medical checklist will be filled out to examine severe physical and mental health problems. In addition, eligibility questions will be answered: age, language proficiency, singleton versus twin pregnancy.
Self-developed delivery questionnaire		x				Infant weight, Apgar, birth and delivery complications, born ≥37 weeks of pregnancy
Demographics						
Demographics questionnaire	x					Maternal age, educational level, SES, drugs use, alcohol use, smoking, number of siblings, age of siblings.
Self-developed delivery questionnaire		x				Infant sex.
Physical contact						
Daily logbook/weekly logbook/questionnaire on physical contact		x(w1–12)			x	Mothers will register 1) holding, 2) SSC, and 3) no contact, as three distinct behavioural categories, for every 15 min with simple lines for the first 5 weeks after birth. In the daily logbook, mothers are able to discriminate between holding and SSC by the mother or other caregivers. Between week 5 and week 12, all mothers will note the estimated time spent in daily holding and SSC on a weekly basis. During the follow-up assessment at 1 year after birth, mothers will indicate how many weeks after week 12 they provided SSC to their infant.
SSC protocol adherence						
Maternal prenatal depressive symptoms, anxiety, and stress	x					The EPDS to screen for depressive symptoms [53]. The Pregnancy-Related Anxieties Questionnaire-Revised (PRAQ-R) is a 34-items validated questionnaire to screen for pregnancy related anxieties [71]. The STAI to screen for anxiety [54]. The Pregnancy Experience Scale (PES) is a 43-items validated questionnaire to assess perceived maternal appraisal of pregnancy-related daily hassles [72]. A 20-item self-developed questionnaire to examine worries related to the preparation for the baby’s arrival. The APL to screen for the occurrence and intensity of daily hassles [55].
Mother-infant bond	x					The Maternal Antenatal Attachment Scale (MAAS) is a 19-item validated questionnaire to assess maternal attitudes, thoughts, and feelings towards the unborn child [73].
Self-developed delivery questionnaire		x				Number of days the father stayed at home after birth.
Weekly logbook sleep location & night awakenings		x(w1–12)				The mother will register the primary sleep location of the infant during 00.00–05.00 h for the previous week [47, 74]. Also, the average number of night awakenings of the infant between 00.00–05.00 h will be noted in the weekly logbook.
Parental ethnographies questionnaire	x		x			10-item questionnaire to assess cultural conceptions on parenting [75].
Social support questionnaire	x		x			Adjusted version of the Social Support Effectiveness Questionnaire (SSE-Q) to examine instrumental, informative and emotional partner support, and negative affect [76].
Social touch			x			The Social Touch Questionnaire (STQ) is a 20-item questionnaire to assess attitudes towards social touch [77].
Adult attachment			x			The Experiences in Close Relationships Scale (ECR) is a validated 36-item questionnaire to asses adult attachment to her current and previous partners [78].

### Study protocol

**Fig. 2:**
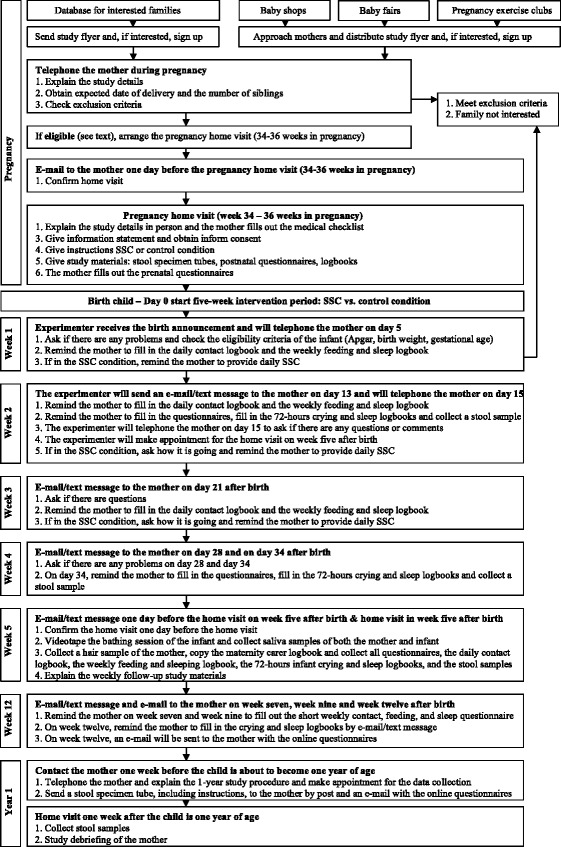
Study procedure

See Table 4 and Fig. 2 for details of the study procedure.

**Table 4:**
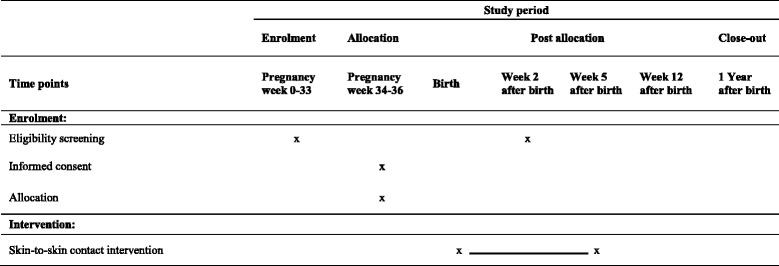
Enrolment and intervention schedule

### Recruitment

The recruitment includes a cover story. Mothers will be told that the study will focus on the relationships between infant sleep and feeding behavior, mother-infant contact, and maternal and infant mental and physical health. In addition, the mothers will be told that a subgroup of the pregnant women will be asked to implement a natural and simple 5-week daily contact period with their newborn infant, starting immediately after birth.

Women will be recruited from a database of pregnant women who have expressed their interest for participating in scientific research. In addition, the experimenter will recruit women at pregnancy clubs, baby fairs, and baby shops. All expectant women will be informed about the study with the study flyer and study website.

After signing up, all expectant mothers will be telephoned by the principle investigator to provide more information about the study. The eligibility questionnaire will assess the eligibility of the mother. Next, a home visit in late pregnancy (week 34–36) will be arranged for interested and eligible women to explain the study in person by the principle investigator.

### Randomization

Primiparous mothers are expected to have more opportunities to provide SSC to their infants compared to multiparous mothers. A stratified random block randomization method will ensure a balance between the SSC and the control condition for the primiparae versus multiparae mothers. Also, this randomization method produces comparable groups by avoiding confounding from known and unknown factors [49]. The block sizes will be randomly chosen from the set of 4 and 6. Randomization will be with a 1:1 allocation. An independent researcher, who is not involved in the study, will prepare this schedule by a computerized randomisation program and will conceal the allocation sequence in stapled envelopes. The allocation sequence will be concealed from the researcher enrolling and assessing participants. The mothers will be included in the study after signing the informed consent, regarding the use of participant data and biological samples (see Additional file 3 for the Dutch versions of the informed consent form and information statement for participants), and filling out the medical checklist during the pregnancy home visit. Hereafter, the principal investigator will open the envelope and the mothers in the SSC group will be informed about their group allocation. Participants and the principle investigator will not be blinded for the group allocation.

### Intervention and control condition

The study of Bigelow and colleagues requested their mothers to provide at least 6 h of SSC per day in the first week after birth and continue with at least 2 h of SSC per day until the infant was one moth of age [15]. However, many mothers did not meet the SSC criterion. In this study we reduce the amount of requested hours and extend the number of requested weeks of daily SSC to enlarge the number of mothers that meet the SSC criterion. In the Netherlands mothers are entitled to at least 10 weeks of paid maternity leave after delivery, which will facilitate adherence to our adapted SSC protocol. During the instruction phase of the pregnancy home visit, all mothers in the intervention group will be provided with detailed written and oral instructions on SSC. We will request and encourage mothers in the SSC condition to provide their infants with at least one uninterrupted hour of maternal SSC, on a daily basis and for 5 weeks, starting immediately after birth. Other caregivers are allowed to provide supplementary hours of SSC to the infant. The period of one uninterrupted hour is based on two arguments. First, the average duration of the sleep cycle of healthy full-term infants is 47 min [50]. The requested uninterrupted hour decreases the chance that infants need to be disturbed in the middle of a sleep cycle. Second, shorter SSC episodes could potentially decrease or eliminate the effectiveness of SSC, since undressing and dressing can be defined as a mild physical stressor for infants [51]. The requested uninterrupted hour requires to undress and dress the infant only once. During SSC, the naked infant, wearing only a diaper, will be placed in a stable and upright position between the breasts of the mother to facilitate and maximize SSC. The head of the infant is positioned in a slightly upright position to keep the airway open. After, the infant and mother will be covered by a blanket or cardigan (see also the practical guidelines of the World Health Organization [11]). Mothers in the SSC condition will explicitly be asked to feed their child before SSC. However, we will not discourage breastfeeding during SSC. During the oral and written instructions, we will explain safety precautions during SSC. We will emphasize the importance of being awake and alert during SSC, and of avoiding drinking hot beverages during SSC to protect the infant. During the second home visit (week 5 after birth), we will ask mothers whether they experienced problems with the safety precautions. Mothers in the control condition will not be requested and encouraged to provide SSC to their infant.

Mother in both groups will fill out the same daily contact logbook. In the daily logbook, all mothers will be asked to register for every 15 min with simple lines the following three categories: 1) holding, 2) SSC, and 3) no contact. In this logbook, mothers are also able to discriminate between holding and SSC by the mother or other caregivers, for example the father or grandparents.

Upon signing the informed consent, participants are informed that they may decide to discontinue the study at any moment and without giving a reason. Several measures are taken in order to minimize attrition and maintain adherence to the study. During the 5-week trial period, the principal investigator will have weekly contact with the participating mothers. Participants will be reminded of the upcoming data collection and they will be asked about any problems they are having with skin-to-skin contact, in filling out the logbooks, or with the collection of the stool samples. In addition, to improve participant retention the mothers will receive a small present after birth, and a present and a financial reimbursement of 20 euros at the home visit in week 5. Last, the mothers will receive the study-wide results after publication of the study reports.

### Pregnancy home visit

One day before the pregnancy home visit, an e-mail will confirm the home visit. During this visit, the researcher reconfirms eligibility. Based on the cover story, the experimenter will provide additional information about the study. If mothers are still interested, informed consent is obtained by the principle investigator and mothers will fill out the medical checklist. Next, the SSC group will be informed about the group allocation. Expectant mothers in the SSC group will receive detailed instructions on SSC. All expectant mothers will receive the information statement with a summary of the instructions about the prenatal and postnatal part of the study. Also, the experimenter will explain how the study logbooks and questionnaires need to be filled out, and how the samples need to be collected. Additionally, the mother will be asked about her preference related to the communication during the intervention period (e-mail versus text-message). Lastly, the mother will fill out the prenatal questionnaires.

### Five-week intervention period starting at birth

The mother will be asked to inform the researcher of the baby’s birth by e-mail, text message or telephone. Five days after birth, the researcher will telephone all mothers to assess the eligibility criterion of the baby and to remind them to fill out the daily contact logbook and the weekly feeding and sleep logbook. Also, the researcher will ask if they have any questions or comments. If the mother is in the SSC condition, the researcher will encourage her to provide daily SSC to the infant.

### Assessment on week two after birth

Thirteen days after birth, all mothers will receive an e-mail or text message to remind them to fill out the additional questionnaires, the 72-h infant crying and sleep logbook, the maternal sleep logbook, and to collect an infant stool sample the next day. Fifteen days after birth, all mothers will be telephoned to ask for questions or comments related to the data-collection on week 2. Also, the home visit on week 5 will be arranged, preferably 4 days after the infant becomes 5 weeks of age.

### Week three and week four after birth

Twenty-one days and 28 days after birth an e-mail or text message will be sent to the mother to remind her to fill out the daily contact logbook, the weekly feeding and sleep logbooks, and she will be asked for any questions or comments. The mothers in the SSC condition will be encouraged to provide daily SSC to the infant.

### Home visit on week five after birth

On 34 days after birth, the experimenter will remind the mother to fill out the additional questionnaires, the 72-h infant crying and sleep logbook, the maternal sleep logbook, and to collect an infant stool sample on week 5. One day before the expected home visit an e-mail or text message will be sent to the mother to reconfirm the home visit. During the home visit, the mother will bathe her infant as usual. Saliva will be collected from both the mother and infant before and two times (25 and 40 min) after the bathing session. The bathing session will be videotaped to observe the quality of maternal caregiving behavior from tape. Also, the researcher will copy the logbook of the maternity carer from the first week after birth. In addition, a hair sample will be collected from the mother for the assessment of maternal physiological stress levels. The experimenter will collect all study questionnaires, logbooks and samples to take back to the lab.

### Assessment on week twelve after birth

Between week 5 and week 12, the mother will be reminded on week 7 and week 9 to fill out the short weekly questionnaire on infant contact, feeding, and sleep by e-mail or text-message. On week 12, the experimenter will send an e-mail to the mothers to remind her to fill out the digital version of the additional questionnaires and to fill out the 72-h infant crying and sleep logbook and the maternal sleep logbook. Afterwards, the mothers will send the logbooks and a copy of the growth curve of the well-baby clinic by post.

### Follow-up assessment on one year after birth

One week before the child’s first birthday, all mothers will be telephoned to explain the study procedure on 1 year after birth and the experimenter will send a birthday card and information related to the stool sample to the mother. Also, a digital version of the additional questionnaires will be sent to the mothers by e-mail. Afterwards, the experimenter will visit the home to collect a stool sample and copy the infant’s growth curve from the well-baby clinic logbook. During this short visit, the mother will also receive the debriefing of the study.

### Data storage

All information will be stored with ID code numbers to maintain participant confidentiality. The ID code numbers will be unrelated to participants’ identifiers, except in a central file with the participants’ contact details. All records that contain names or other personal identifiers, such as informed consent forms, will be stored separately from study records identified by the ID code number. All study-related information on paper, including logbooks, questionnaires, and administrative forms, and the maternal hair samples will be securely stored in locked file cabinets in areas with limited access. Biological specimens, including maternal and infant saliva samples and infant stool samples, will be stored in medical refrigerators in the laboratory area, with limited access, of the Behavioural Science Institute. Digital and online information will be secured with password-protected access systems. Two investigators will check the imported data values before the data analyses. Only the principal investigators will be given full access to the data. The Behavioural Science Institute is currently working on an open access database. The entire dataset of this study will be included in this database after publication of all study reports. The dataset will only be shared without participant identifiers.

### Data-analysis

Data will be handled according to standard procedures. Demographic characteristics and study outcomes will be described for each treatment group using means and standard deviations for continuous outcomes. Proportions for categorical data will be provided. The pattern of missingness will be checked before the analyses. All measures will be controlled for outliers and checked for normality. Skewed continuous outcomes will be transformed. The level of significance (*p* -value) is set at *p* < .05 in all analyses.

We will conduct intention-to-treat analyses, in which participants are compared according to the group they were randomly assigned to regardless of participants’ compliance, including SSC protocol adherence, or withdrawal from the study, and analyses on imputed datasets based on the multiple imputation method [52]. Primary and secondary outcomes, that will be examined once, will be compared within multivariate analyses of covariance. Multilevel mixed models will be used for repeated measures. Microbiota composition will be examined with the Pearson’s moving window correlation. Microbiota diversity will be calculated with the Simpson’s reciprocal index of diversity (1/D). The Benjamini and Hochberg method will analyse differences between the SSC group and the control group. The analyses will be adjusted for potential confounding factors identified a priori (see Table 3). Structural equation models will be used to examine the mediation effect of the potential underlying mechanisms, maternal oxytocin concentrations and infant intestinal microbiota, on maternal and infant outcomes, respectively.

### Dissemination

Data will be analysed when all the data is collected. The principal investigators will make every attempt to reduce the interval between the completion of data collection and the release of study results to a minimum. The principle investigators will justify names for authorship regarding topics suggested for presentation or publication. Data will only be reported study-wide. The study results will be released in a study-wide manner to participants after publication of the results. Also, the study results will be released to the general public by several channels, including international high impact journals and, at a national level, by oral presentations in health care organizations to inform nurses, midwifes, pediatricians and mental healthcare practitioners about this intervention method.

## Discussion

Maternal depressive symptoms in the postpartum period are highly prevalent and affect the mother and child in the short-and long-term. Most of these mothers remain untreated for a long period of time or are not treated at all. There is great need for simple and cost-effective prevention and (complementary) intervention methods that are easily accessible to mothers and that can be applied immediately after birth. SSC is such a low-cost intervention that it would be accessible, simple, and feasible for most mothers in the first postpartum weeks.

In preterm infants, SSC has been shown to be beneficial for the mother and the infant in reducing maternal postpartum depressive symptoms. Additionally, the preterm infant grows quicker and is healthier, cries less and sleeps better, while mothers are less anxious and stressed, invest more in breastfeeding, and feel more bonded to their infant. SSC may be a promising method for mothers and their full-term infants as well. To date, most studies have focused on preterm infants or on SSC in full-term infants during the first postnatal hours. If SSC is shown to be effective in reducing depressive symptoms in low-risk mothers of infants born full-term, then an SSC intervention could potentially be investigated in high-risk samples of prenatally and postnatally depressed mothers who should be carefully screened (e.g. for touch aversion) and closely monitored and supported during the intervention period. Also, thought could be given to developing programs using SSC in a preventive manner. SSC may also constitute a cost-effective prevention program for mothers and their children enhancing and strengthening developmental processes. Finally, by exploring two possible underlying mechanisms of SSC, this study will additionally provide important and innovative scientific knowledge that will deepen our understanding of the biology underlying mother-infant skin-to-skin contact and dyadic interactions.

## Additional files

Additional file 1: SPIRIT 2013 Checklist. The complete SPIRIT 2013 checklist. (PDF 220 kb)

Additional file 2: Administrative information. Administrative information related to: (1) title; (2) the online trial registration; (3) the protocol version; (4) the funding sources; (5) the roles and responsibilities of the protocol contributors and the trial funders. (PDF 241 kb)

Additional file 3: Informed consent form & information statement. Dutch versions of the Informed Consent form and the Information Statement for participants. (PDF 666 kb)

## Abbreviations

APL: Alledaagse Problemen Vragenlijst
ASQ-3: Ages & stages questionnaire, third edition
AUC: Area under the curve
BISQ: Brief infant sleep questionnaire
BITSEA: Brief infant toddler social emotional assessment
ECR: Experiences in close relationships
EPDS: Edinburgh postnatal depression scale
HPA: Hypothalamic-pituitary-adrenal
IBQ-R: Infant behavior questionnaire revised
KSD: Karolinska sleep diary
MAAS: Maternal antenatal attachment scale
MFI: Multidimensional fatigue inventory
MPAS: Maternal postnatal attachment scale
NTR: Dutch trial registration
PES: Pregnancy experience scale
PRAQ-R: Pregnancy-related anxieties questionnaire revised
PSQI: Pittsburgh sleep quality index
RCT: Randomized controlled trial
SF-36: Short-Form Health Survey
SSC: Skin-to-skin contact
SSE-Q: Social support effectiveness questionnaire
STAI: State-trait anxiety inventory
STQ: Social touch questionnaire
TES-B: Traumatic event scale – B
