# Supplementary material for: Effectiveness of skin-to-skin contact versus care-as-usual in mothers and their full-term infants: study protocol for a parallel-group randomized controlled trial
Source: BMC Pediatr. 2017 Jul 6;17:154. doi: 10.1186/s12887-017-0906-9 (PMC5501342; doi:10.1186/s12887-017-0906-9)
Supplement: Supplementary file 2 — Administrative information. Administrative information related to: (1) title; (2) the online trial registration; (3) the protocol version; (4) the funding sources; (5) the roles and responsibilities of the protocol contributors and the trial funders. (PDF 241 kb) [file 12887_2017_906_MOESM2_ESM.pdf]

Additional file 2

1. Administrative information about the trial:

1. Title
2. Online trial registration
3. Protocol version
4. Funding sources
5. Roles and responsibilities of the protocol contributors and trial funders

Administrative information

1. Title

Effectiveness of skin-to-skin contact versus care-as-usual in mothers and their full-term infants: Study protocol for a parallel-group randomized controlled trial

2. Trial registration

Primary registry and trial identifying number	Dutch Trial Registration<br>NTR5697
Date of registration in primary registry	2016-March-13
Secondary identifying numbers	Ethics Committee Social Sciences; Radboud University<br>ECSW2015-2311-358
Sponsor/Initiator	Radboud University and the Behavioural Science Institute
Funding	Radboud University and the Behavioural Science Institute
Contact for public queries	Kelly Cooijmans, MSc.<br>Radboud University<br>Developmental Psychology<br>Montessorilaan 3<br>6525 HP Nijmegen<br>Tel. +31-24-361 2658<br>Mail: k.cooijmans@psych.ru.nl
Contact for scientific queries	Kelly Cooijmans, MSc.<br>Radboud University<br>Developmental Psychology<br>Montessorilaan 3<br>6525 HP Nijmegen<br>Tel. +31-24-361 2658<br>Mail: k.cooijmans@psych.ru.nl
Public title	Skin-to-skin contact in mothers and their full-term infants
Scientific title	Effectiveness of skin-to-skin contact versus care-as-usual in mothers and their full-term infants: Study protocol for a parallel-group randomized controlled trial
Health conditions and problems studied	Mothers who just gave birth to their child.
Inclusion criteria	Mothers who just gave birth to their child;<br>Aged ≥ 18;<br>Singleton pregnancy;<br>Infant born at ≥ 37 weeks of pregnancy;<br>Infant birth weight ≥ 2500 gram;<br>Infant ≥7 5-min Apgar score;<br>Exclusive participation in this intervention trial.
Exclusion criteria	Drug use during pregnancy;<br>Severe maternal physical or mental health problems;<br>Insufficient understanding of Dutch;<br>Congenital anomalies.
Study type	Interventional (intervention versus passive control group)<br>Allocation: randomized<br>Intervention model: two-arm parallel assignment<br>Masking: no blinding

Intervention	Mothers in the skin-to-skin contact condition will be requested and encouraged to provide at least one daily and continuous hour of skin-to-skin contact to their infant for the first 5-weeks after birth. The control group will not be requested and encouraged to provide daily skin-to-skin contact to their infant. Both groups will fill out the same logbooks and questionnaires, will collect the same samples and will perform the same tasks.
Planned date of enrolment	2016-April-01
Country of recruitment	The Netherlands
Recruitment status	Recruiting
Target sample size	116 (58 participants in each group)
Primary outcomes	*Improved maternal outcomes*<br>1. Mental health:<br>• Depressive symptoms
Secondary outcomes	*Improved maternal outcomes:*<br>1. Mental health:<br>• Anxiety;<br>• Stress;<br>• Traumatic stress following child birth;<br>• Sleep quality.<br><br>2. Physical health:<br>• Physical recovery from to the delivery;<br>• Health (illnesses and health problems);<br>• Breastfeeding duration and frequency;<br>• Physiological stress.<br><br>3. Mother-infant relationship:<br>• Bonding to the infant;<br>• Quality of maternal caregiving behavior.<br><br>*Improved infant outcomes:*<br>1. Behavior:<br>• Fussing and crying;<br>• Sleep quality.<br><br>2. Physical health:<br>• Growth and health (illnesses and health problems);<br>• Physiological stress.<br><br>3. General development:<br>• Regulation capacities;<br>• Social-emotional capacities;<br>• Language, cognitive, and motor capacities.<br><br>*Potential underlying mechanisms:*<br>1. Maternal oxytocin concentrations<br>2. Infant intestinal microbiota

3. Protocol version

**Revision chronology**	
2016-June-22	Original manuscript
2016-July-31	Amendments:<br>1. We now included the trial registration date in the Abstract of the manuscript (page 3, line 7).<br>2. We now added statements to the protocol to better clarify and define the statement: "A 5% significance level and a power of 80% was used for the analysis" (page 13, line 12-17).<br>3. We now included a reference for the statement: "Based on prior studies in similar populations…" (page 13, line 17).<br>4. To adhere to the journal guidelines we now moved the "List of Abbreviations" and the "Declarations" to the end of the manuscript, after the Discussion/Conclusions (page 23-25).<br>5. To adhere to the journal guidelines we now included all sections of the Declarations (page 24-25).<br>6. To adhere to the journal guidelines we now moved the "Administrative information" summary to "Supplementary file 2".<br>7. We now included a "Supplementary files" section after the references with a list of all file names, titles and a description of every file.
2017-May-19	Amendments:<br>1. We now consequently use "full-term infants" and "preterm infants" throughout the manuscript instead of "fullterms" and "preterms" (see page 2-7).<br>2. We now consequently included "depressive symptoms" instead of "depression" in the manuscript when the word "depression" was erroneously used (see page 3, 5, 6, 9, 10, 38, 40).<br>3. We deleted the implication that SSC and KMC are the same (page 4, line 5).<br>4. We now rewrote the statements on page 6, line 6-8, to better clarify the significance level of study #15 and we included the sample sizes of this study.<br>5. We now better specify the pathways of the potential underlying mechanisms (page 9, line 4).<br>6. We now included a statement to better clarify how we will assure equal groups on key variables (see page 15, line 20-21).<br>7. We now included more detailed information on the SSC protocol. The study protocol now explicitly includes:<br>• information on the paid maternity leave after delivery in the Netherlands to better clarify the feasibility of the requested hour of SSC (see page 16, line 15-16).<br>• that mothers will be asked to provide their infants with at least one uninterrupted hour of maternal SSC, on a daily basis and for five weeks, starting immediately after birth (see page 16, line 18-21).<br>• that other caregivers are allowed to provide supplementary hours of SSC to the infant (see page 16, line 20-21).<br>• two references that explain why we chose to ask for an uninterrupted, instead of cumulative, hour of SSC (see page 16, line 21-25, & page 17, line 1-2).<br>• that all mothers in the SSC condition, will explicitly be asked to feed their child before SSC. However, we will not discourage breastfeeding during SSC since SSC stimulates breastfeeding (see page 17, line 7-8).

	• that we will explain safety precautions during SSC. We will emphasize the importance of being awake and alert during SSC, and of avoiding drinking hot beverages during SSC to protect the infant. We will now personally ask the mother about problems in safety precautions during the second home visit on week five after birth (see page 17, line 8-12).<br>• that mothers in both groups will fill out the same daily contact logbook (see page 17, line 14).<br>• that all mothers will be asked to register for every 15 minutes with simple lines the following three categories: 1) holding, 2) SSC, 3) no contact (see page 17, line 14-16, & Table 3).<br>• that all mothers are able to discriminate between holding and SSC by the mother or other caregivers, for example the father or grandparents (see page 17, line 16-18, & Table 3).<br>8. We now explicitly included "SSC protocol adherence" in the manuscript, to better clarify the intention-to-treat analyses (see page 21, line 22).<br>9. We modified the section on the potential use of SSC in high-risk samples. We now emphasized the importance of screening (e.g. for touch aversion) and monitoring high-risk mothers during the intervention period (see page 23, line 11-13).<br>10. We now added statements to the manuscript to better clarify and define "cortisol synchrony" (see Table 1).<br>11. We adapted the lay-out of Table 3 to better clarify the difference between eligibility criteria, demographics, physical contact information and variables that will be collected to gain insight into the SSC protocol feasibility and protocol adherence.<br>12. We added information on the primary sleep location (Table 3).<br>13. We will collect the maternal age and maternal education level but we had not described this information in the study protocol. We have now included these variables in the manuscript (Table 3).<br>14. We now explicitly added that the Experiences in Close Relationships (ECR) questionnaire examines the mother's attachment to her current and previous partners (see Table 3).

4. Funding

The Radboud University, The Netherlands, and the Behavioural Science Institute, The Netherlands, funded the project with an internal grant.

5. Roles and responsibilities of the protocol contributors and trial funders

5.1 Authors' contributions

KHMC was involved in the design of the study, in drafting the manuscript and will be the principal investigator of the study. RB was involved in the study design and critically revised the manuscript for important intellectual content. ACR was involved in the study design and wrote the first draft of the study manuscript. CdW developed the original concept, was involved in the study design and critically revised the manuscript for important intellectual content. All authors read and approved the final manuscript.

5.2 Sponsor contact information

Trial sponsor:	Radboud University & Behavioural Science Institute
Sponsor Reference:	N/A
Contact name:	Ms M. van den Eng
Address:	Behavioural Science Institute<br>Radboud University<br>P.O. Box 9104<br>6500 HE Nijmegen<br>The Netherlands
Telephone:	+31-24-361 0082
Email:	secr@bsi.ru.nl

5.3 Sponsor and funder

The Radboud University, The Netherlands, and the graduate school of the Behavioural Science Institute, The Netherlands, had no role in the design of this study and will not have a role during its execution, analyses, interpretation of the data, or decisions related to the submission of results.

5.4 Committees

Committee	Description
Investigators	Design of the study; Conducting the study; Preparation of the protocol and revisions; Preparation materials; Study planning; Recruitment of participants; Budget administration; Data collection; Organisation of sample collection; Data entry; Publication of study results.
Independent researcher	Preparation randomization sequence; Concealment of the allocation sequence in stapled envelopes.
PhD committee of the Behavioural Science Institute	Monitor study planning and study progress.
