# Supplementary material for: Effectiveness of skin-to-skin contact versus care-as-usual in mothers and their full-term infants: study protocol for a parallel-group randomized controlled trial
Source: BMC Pediatr. 2017 Jul 6;17:154. doi: 10.1186/s12887-017-0906-9 (PMC5501342; doi:10.1186/s12887-017-0906-9)
Supplement: Supplementary file 3 — Informed consent form & information statement. Dutch versions of the Informed Consent form and the Information Statement for participants. (PDF 666 kb) [file 12887_2017_906_MOESM3_ESM.pdf]

Additional file 3

1.Dutch version of the Informed Consent form

2.Dutch version of the Information Statement for participants

Radboud Universiteit

Dutch version of the Informed Consent form

TOESTEMMINGSVERKLARING

voor deelname aan het wetenschappelijk onderzoek:

SKIPPY onderzoek

Door deze pagina te ondertekenen stemt u in met het volgende:
• Ik ben naar tevredenheid over het onderzoek geïnformeerd.
• Ik heb de schriftelijke informatie goed gelezen.
• Ik heb begrepen dat de verzamelde data alleen voor onderzoeksdoeleinden gebruikt worden.

Dit houdt in dat:

- ik ben in de gelegenheid gesteld om vragen over het onderzoek te stellen. Mijn vragen zijn naar tevredenheid beantwoord.
- ik heb goed over deelname aan het onderzoek kunnen nadenken
- het verzamelde materiaal anoniem onder een codenaam bewaard wordt (losgekoppeld van de naam en personalia van mijzelf en mijn kind)
- het verzamelde materiaal alleen toegankelijk is voor de betrokken onderzoekers
- het verzamelde materiaal na 20 jaar wordt vernietigd
- er geen menselijke genetische informatie uit het verzameld lichamelijk materiaal gehaald zal worden. Lichamelijk materiaal houdt in: speeksel van mijzelf en mijn kind, haar van mijzelf, en ontlasting van mijn kind.
- ik heb het recht mijn toestemming op ieder moment weer in te trekken zonder dat ik daarvoor een reden behoef op te geven
- ik begrijp dat ik mijn vragen altijd kan richten tot de onderzoeker: Kelly Cooijmans, skippy@psych.ru.nl, telefoonnummer: 024 361 2658.

Ik geef toestemming aan de leerstoelgroep Ontwikkelingspsychologie om het tijdens dit onderzoek opgenomen videomateriaal te tonen (kruis aub één van onderstaande opties aan):

- o Ter illustratie van het onderzoek (aan vakgenoten)
- o Ten behoeve van onderwijsdoeleinden (aan studenten)
- o Ter illustratie van het onderzoek en ten behoeve van onderwijsdoeleinden
- o Ik geef geen toestemming om opgenomen videomateriaal te tonen

Kruis aub één van onderstaande opties aan. Met uw handtekening geeft u aan het met bovenstaande uitspraken eens te zijn.

- o Ik stem toe met deelname aan het onderzoek.
- o Ik stem niet toe met deelname aan het onderzoek.

Naam :……………………………………………………………………………………………
Geboortedatum: ……-……-……
Handtekening :………………………………………………………. Datum: ….….-……..-…….

Ondergetekende verklaart dat de hierboven genoemde persoon zowel schriftelijk als mondeling over het bovengemelde onderzoek geïnformeerd is. Zij verklaart tevens dat een voortijdige beëindiging van de deelname door bovengenoemde persoon, voor haar verder geen gevolgen heeft.
Naam :……………………………………………………………………………………………
Functie:…………………………………………………………………………………………..
Handtekening :………………………………………………………. Datum: ….….-……..-……..

Dutch version of the Information Statement for participants

Geachte mevrouw,

Wij zijn erg blij dat wij u mogen verwelkomen als nieuwe deelnemer van het SKIPPY-onderzoek! Vandaag heeft u een kennismakingsgesprek gehad met de onderzoeker van SKIPPY. In deze informatiebrief zal datgene wat besproken is nogmaals worden beschreven, zodat u rustig alles nog eens kunt nalezen. Mocht u nog vragen hebben dan kunt u met al uw vragen terecht bij de onderzoeker Kelly Cooijmans (telefoon: 024 361 2658 / mobiel: 06 11240533 / email: skippy@psych.ru.nl).

Waarom is dit onderzoek belangrijk?

1. In dit onderzoek willen wij kijken hoe slaap, voeding en contact tussen moeder en haar pasgeboren baby gerelateerd zijn aan zowel de ontwikkeling en gezondheid van de baby, als het herstel en welzijn van de moeder.
2. Ook willen wij graag kijken of een aantal tot nu toe weinig bestudeerde biologische factoren ons meer kunnen vertellen over deze eerdergenoemde variabelen. Zo gaan wij kijken naar de darmbacteriën van de baby, welke belangrijk zijn voor een goede gezondheid en de ontwikkeling van het immuunsysteem. Ook willen wij gaan kijken naar de hormonen van zowel de baby als de moeder.
3. Aan een subgroep van de moeders vragen wij om op een natuurlijke en eenvoudige manier dagelijks contact met de baby te hebben gedurende de eerste vijf weken na de geboorte. Op deze manier willen wij kijken of dit contact de gezondheid en het herstel van zowel de baby als de moeder op een eenvoudige manier kan bevorderen.

Overzicht van het onderzoek

Leeftijd van uw baby	Taak
Huisbezoek tussen 34-36 weken zwangerschap	• Kennismakingsbezoek en uitleg over de studieprocedure<br>• Tekenen toestemmingsformulier
Eerste 5 weken na geboorte	• Na de geboorte **start** u met het invullen van een eenvoudig dagelijks contact logboek en een wekelijks logboek over slaap en voeding<br>• Een subgroep van de moeders zal dagelijks op een eenvoudige en natuurlijke manier contact hebben met de baby
Week 2 & week 5	• Vragenlijsten invullen<br>• Drie dagen een slaap en gedragslogboek invullen<br>• Ontlasting baby verzamelen
Huisbezoek week 5	• Na 5 weken **stopt** het invullen van het dagelijks logboek<br>• Thuisbezoek (badje geven aan de baby)<br>• Speeksel samples van moeder en kind tijdens huisbezoek rondom badje<br>• Haar sample moeder tijdens huisbezoek
Week 5 t/m 12	• Wekelijks een klein aantal vragen invullen
12 weken	• Online vragenlijsten invullen<br>• Drie dagen een slaap en gedragslogboek invullen
1 jaar	• Online vragenlijsten invullen<br>• Ontlasting baby verzamelen

1. Bezoeken

Het onderzoek bestaat uit twee huisbezoeken. Het eerste bezoek heeft u vandaag gehad. Zojuist is een SKIPPY-onderzoeker bij u op bezoek geweest om kennis met u te maken en om u meer te vertellen over de studie procedure na de geboorte van uw baby. Tijdens dit bezoek is ook kenbaar gemaakt of u een deelnemer bent van de subgroep.

Het tweede thuisbezoek zal plaatsvinden als uw baby 5 weken oud is. Dit bezoek zal ongeveer 1.5 uur duren. Tijdens dit bezoek zal u uw baby in bad doen, zoals u dat normaal ook doet. Wij zullen drie keer speeksel bij u en uw baby verzamelen. Het geven van het badje zal gefilmd worden.

2. Vragenlijsten

Gedurende de eerste vijf weken na de bevalling houdt u dagelijks een logboek bij, door middel van simpele lijnen, over contact met uw kind. Daarnaast zullen wij u vragen om wekelijks een klein aantal vragen in te vullen voor de eerste twaalf weken. Als uw baby 2, 5, 12 weken en 1 jaar oud is, zullen wij u vragen om een aantal vragenlijsten in te vullen over het gedrag en de gezondheid van uw baby en uw eigen herstel en gezondheid. Ook zouden wij graag het logboek van de kraamverzorging over willen nemen tijdens het bezoek op 5 weken en willen wij u vragen om de groeicurve van uw baby met ons te delen op 12 weken en 1 jaar.

3. Biologische factoren van uzelf en uw kind

U zult gevraagd worden om op drie momenten na de bevalling (op 2 en 5 weken, en op 12 maanden) de ontlasting van uw kind te verzamelen (uit de luier). Ook zouden wij graag tijdens het thuisbezoek een kleine hoeveelheid haar van u verzamelen om te kijken hoe hormonen veranderen gedurende de zwangerschap en in de periode daarna. Mocht u liever geen haar afstaan, dan is dat geen probleem. Wel willen wij benadrukken dat het knippen van het haar geen pijn doet en dat het om een zeer kleine hoeveelheid haar gaat waar je niets van ziet.

Mogelijke exclusie na de geboorte van de baby

Binnen het onderzoek hanteren wij een aantal criteria voor zowel de moeder als de baby. Op basis van deze criteria kunnen wij bepalen welke moeders en baby's mee kunnen doen aan het onderzoek. De criteria van de moeder zijn gecontroleerd tijdens de zwangerschap. Voor het controleren van de criteria van de baby zullen wij op dag 5 na de geboorte telefonisch contact met u opnemen. Voor deelname is het belangrijk dat:

- Uw kind geboren wordt ná 37 weken zwangerschap
- Uw kind minimaal 2500 gram weegt bij geboorte
- Uw kind bij geboorte een 5-minuten APGAR-score heeft van ten minste 7
- Uw kind geen geboorteafwijkingen heeft

Voor- en nadelen voor de deelnemer

Een nadeel voor u is dat het onderzoek u extra tijd kost door het invullen van de logboeken, de vragenlijsten en het verzamelen van materialen (ontlasting baby). Een voordeel voor u is dat u bijdraagt aan wetenschappelijk onderzoek, welke nuttige wetenschappelijke gegevens kan opleveren voor toekomstige ouders waardoor u baby's en moeders in de toekomst kan helpen. Ook kan dit onderzoek ervoor zorgen dat u zich meer bewust wordt van de ontwikkeling en gezondheid van uw eigen kind. Daarnaast zouden de deelnemers in de subgroep mogelijk positieve effecten kunnen ervaren met betrekking tot voeding, gedrag en gezondheid van zowel zichzelf als de baby.

Vergoedingen

Als dank voor uw deelname aan dit onderzoek:

- Ontvangt u een irischeck t.w.v. 20 euro
- Hebben wij leuke cadeautjes voor uw kindje
- Geven wij u een dvd van de opname van uw kindje op 5 weken
- Na afloop van het onderzoek zult u de onderzoeksresultaten ontvangen
- Nieuwsbrieven

Wat gebeurt er als u niet meer wilt deelnemen?

Deelname aan dit onderzoek is geheel vrijwillig. U kunt uw toestemming te allen tijde zonder opgave van redenen weer intrekken. De onderzoeker zal u wel vragen naar de redenen voor u om te stoppen. U hoeft daarbij geen reden op te geven.

Anonimiteit

De verzamelde gegevens zullen volledig anoniem blijven, en worden uiterst voorzichtig behandeld. Dit houdt in dat:

- Uw naam niet gekoppeld wordt aan de verkregen gegevens.
- De gegevens uitsluitend voor onderzoeksdoeleinden gebruikt worden.
- De gegevens alleen toegankelijk zijn voor de betrokken onderzoekers.
- De gegevens veilig bewaard worden voor een periode van 20 jaar, en daarna worden vernietigd.
- Geen menselijk genetische informatie uit de verzamelde lichamelijke materialen verkregen wordt.

Hoe te handelen bij klachten?

Als u klachten heeft over het onderzoek, kunt u dit melden aan de onderzoeker Kelly Cooijmans, tel. 024 361 2658 of email: skippy@psych.ru.nl. U kunt ook contact opnemen met de hoofdverantwoordelijke van het onderzoek, Prof. dr. Carolina de Weerth, te bereiken via telefoon: 024 361 2637 of email: c.deweerth@psych.ru.nl.

Wilt u nog iets weten?

Voor vragen kunt u terecht bij de onderzoeker:

Contact: Kelly Cooijmans
Email: skippy@psych.ru.nl
Telefoon: 024 361 2658
Mobiele telefoon: 06 11240533
Contactadres: Radboud Universiteit Nijmegen
Montessorilaan 3
6525 HR Nijmegen
Website: www.skippyonderzoek.org

Alvast hartelijk bedankt namens het onderzoeksteam!

SKIPPY - onderzoeksteam

Kelly Cooijmans, MSc.
Dr. Roseriet Beijers
Prof. dr. Carolina de Weerth
